# Hemosiderin laden macrophages and hemosiderin within follicular cells distinguish benign follicular lesions from follicular neoplasms

**DOI:** 10.4103/1742-6413.45193

**Published:** 2009-01-19

**Authors:** Reema Jaffar, Sambit K. Mohanty, Ashraf Khan, Andrew H. Fischer

**Affiliations:** Department of Anatomical Pathology, University of Massachusetts, MA USA

**Keywords:** Benign colloid nodule, follicular cells, hemosiderin laden macrophages, hemosiderin within follicular neoplasms, macrophages

## Abstract

**Background:**

Published criteria to distinguish benign colloid nodules from follicular neoplasms emphasize only three interdependent features: size of follicles, amount of colloid, and cellularity. There is a need for the validation of other independent criteria.

**Methods:**

This study quantified the significance of cystic change, defined as presence of macrophages, and the presence of hemosiderin in either the macrophages or follicular cells. The cohort consisted of 165 patients with fine needle aspiration (FNA) and histologic follow-up of either goiter (101), follicular adenoma (47), or follicular carcinoma (17). Papillary thyroid carcinomas and Hürthle cell neoplasms were excluded from the cohort, because these categories are known to show cystic change and hemosiderin. FNAs were reviewed blindly with the most cellular slide scored for the presence of macrophages and/or hemosiderin.

**Results:**

Hemosiderin within macrophages were seen in 67% (68 of 101) of the goiters and only 6% (four of 64) of follicular neoplasms (*P*<.0001). All four follicular neoplasms with hemosiderin in macrophages were adenomas. Three of these four had equivocal features of a benign colloid nodule histologically. None of the 17 follicular carcinomas had hemosiderin in macrophages (*P*<.12). Macrophages without hemosiderin also strongly distinguished goiters from neoplasms (83% vs 17%) but appears less useful as a criterion since macrophages were present within 3 of 17 follicular carcinomas. Hemosiderin within follicular epithelial cells was present in 18% (18 of 101) of goiters, whereas none of the 64 follicular neoplasms had intraepithelial hemosiderin (*P*<.0003).

**Conclusions:**

If papillary thyroid carcinoma and Hürthle cell neoplasm are ruled out, our findings indicate that the presence of hemosiderin virtually excludes a clinically significant follicular neoplasm.

## INTRODUCTION

Fine needle aspiration (FNA) is a rapid, cost-effective, safe, and simple means of diagnosing thyroid nodules.[1–3] Thyroid nodules can be due to a large number of causes, the most common of which is a benign colloid nodule.[4] The main differential diagnosis of benign colloid nodule includes follicular neoplasms (adenoma and carcinoma, of either follicular or Hürthle cell types) and papillary thyroid carcinoma. Hürthle cells are distinguished from usual follicular cells by the presence of abundant granular cytoplasm, reflecting the presence of large numbers of mitochondria within the cytoplasm.[5] Papillary thyroid carcinoma (PTC) is distinguished from benign colloid nodules and follicular neoplasms, mostly on the basis of fine dispersed chromatin and irregular nuclear shape, including elongation, longitudinal nuclear grooves, and intranuclear cytoplasmic inclusions.[6–8]

The current criteria for distinguishing follicular neoplasms from benign colloid nodules have stressed microfollicular architecture, scant colloid and high cellularity relative to colloid content.[9,10] These three features are necessarily interdependent, because a microfollicular arrangement necessarily makes the amount of colloid low, relative to the number of follicular cells. Validation of additional diagnostic discriminators between benign colloid nodules and follicular neoplasms would, therefore, be useful.

Papillary thyroid carcinomas have a tendency to bleed into themselves, perhaps because of twisting and infarction of delicate fibrovascular cores of papillary fragments.[5] About 13% can also be cystic[11] and probably a larger percentage can have some hemosiderin or macrophages. Hürthle cell neoplasms, especially those over three centimeters may exhibit hemorrhage and hemosiderin, and occasionally undergo cystic change.[6] Cystic change and hemorrhage have been suggested to distinguish benign colloid nodules from follicular neoplasms of non-Hürthle type,[6] but we are unable to find published validation of these criteria. We quantified the prevalence of cystic change and hemosiderin in FNAs that had a follow-up histologic diagnosis of either benign goiter or follicular neoplasm and found that regardless of whether a lesion is macro or microfollicular, the presence of hemosiderin or macrophages strongly supports the diagnosis of a benign colloid nodule, provided that PTC or Hürthle cell neoplasm is excluded. The implication of hemosiderin and cystic change for the pathogenesis of benign colloid nodules is discussed.

## MATERIALS AND METHODS

This retrospective study conducted at our institute included a cohort of 165 patients with histopathologically proven cases of benign goiter (*n* = 101), follicular adenoma (FA) (*n* = 47), and follicular carcinoma (FC) (*n* = 17) that had a preceding adequate FNA. PTC and hürthle cell neoplasms were excluded from analysis in this study. None of the patients had undergone a second FNA within the preceding six months, and, thus, any hemosiderin detected would be unlikely due to a previous FNA. The FNAs were performed by the same group of endocrinologists, with 22-25 gauge needles. Typically four to six passes were performed per case. A part of the sample from each pass was smeared and the remaining sample was rinsed in CytoRich Red^tm^ (Thermo Shandon, Pittsburgh, PA). The needle rinse was then centrifuged and transferred to PreservCyt^tm^ (Hologic, Bedford, MA) for making a ThinPrep with a cell block, if residual material remained after the ThinPrep.

Observers, who were blinded to the surgical diagnoses, examined one slide. The slide examined was either the ThinPrep slide or a smear, if the smear was more cellular. The presence or absence of macrophages was noted. Each of the macrophages and each follicular group on the one ThinPrep or conventional smear were examined at 400X magnification, to exclude the presence of hemosiderin. This sampling approach appeared reasonable and practical, since macrophages tended to be easy to find, if they were present at all, and the ThinPrep or most cellular smear provided a reasonable size sample of follicular cells to be scrutinized for the presence of hemosiderin, at high magnification. Prussian blue iron staining in a cell block section was used to confirm the presence of hemosiderin. The statistical significance of the observations was evaluated utilizing the Chi-square test with Yates' correction for 2 × 2 table.

## RESULTS

**Figure 1:**
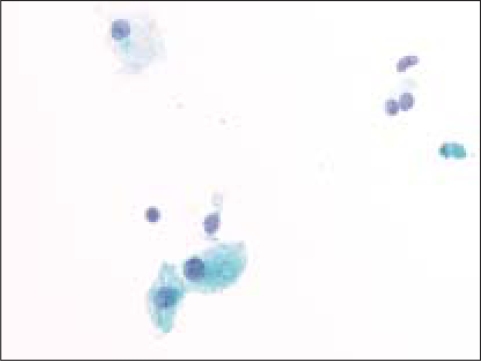
Cystic macrophage in a case of benign colloid nodule (Pap, O.M. ×600)

Macrophages were observed in 83% (84 of 101) of benign goiters [Figure 1]; whereas, only 17% (11 of 64) of the follicular neoplasms had any macrophages in the FNA that preceded the resection (*P*<0.0001) [Table 1]. The eleven cases of follicular neoplasms with macrophages had the following diagnosis on surgical pathology: two follicular carcinomas with lymphovascular invasion, one minimally invasive follicular carcinoma with no lymphovascular invasion, and eight follicular adenomas. Upon review, four of the eight follicular adenomas had histological features equivocal between follicular adenoma and goiter. Another two cases of follicular adenoma had an accompanying diagnosis of lymphocytic thyroiditis.

**Table 1:** Results of the study

*Diagnosis*	*Macrophages (%)*	*Hemosiderin in macrophages (%)*	*Hemosiderin in follicular cells (%)*
Benign goiter (n = 101)	84 (83.2)	68 (67.3)	18 (17.8)
Follicular adenoma (n = 47)	8 (17.0)	4 (8.5)	0
Follicular carcinoma (n = 17)	3 (17.6)	0	0

**Figure 2:**
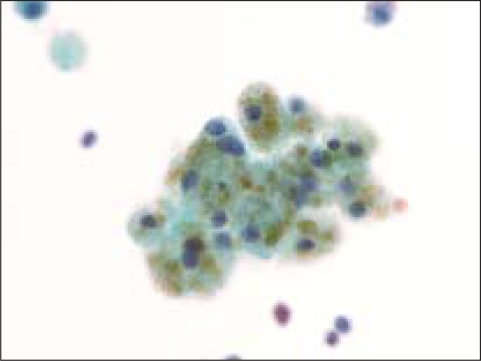
Hemosiderin-laden macrophage in a case of benign colloid nodule (Pap, O.M. ×600)

**Figure 3:**
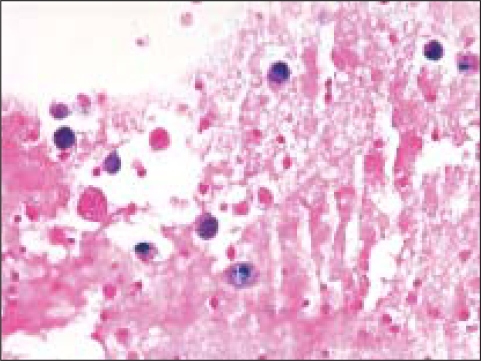
Prussian blue staining demonstrates hemosiderin in macrophages (Pap, O.M. ×400)

**Figure 4:**
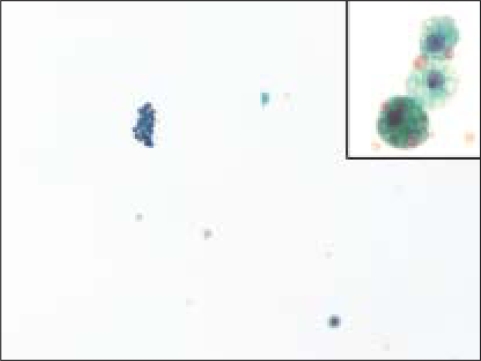
Macrophages were rarely seen in FNA samples that were ultimately diagnosed on resection as a follicular neoplasm. The insert shows a higher magnification of a macrophage from this case, containing hemosiderin (Pap, O.M. ×200; insert is ×600)

**Figure 5:**
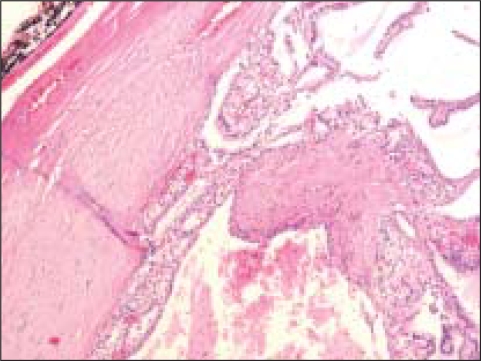
The histologic section from the lesion corresponding to Figure 4 was diagnosed by surgical pathologists as a ‘macrofollicular adenoma’. Note the presence of occasional macrophages within some of the enlarged follicles (Pap, O.M. ×100)

Hemosiderin was present within the macrophages in 67% (68 of 101) cases of benign goiters [Figure 2, Figure 3] and in only 6% (4 of 64) of follicular neoplasms [Figure 4], all of which were follicular adenomas (*P*<0.0001). Upon review, three of the four follicular adenomas had histological features equivocal with a goiter [Figure 5]. No follicular carcinoma had HLM (*P*<0.1247 when compared to FA).

**Figure 6:**
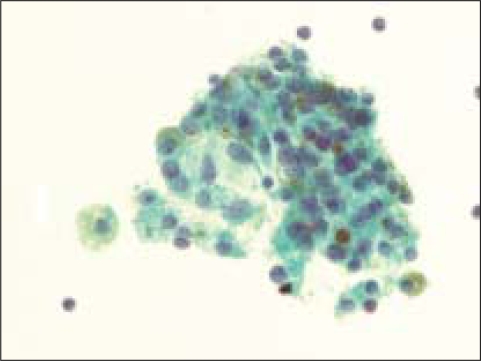
Follicular epithelial cells with hemosiderin granules in a case of benign colloid nodule (Pap, O.M. ×600)

Hemosiderin was seen within follicular epithelial cells in 18% (18 of 101) of the benign goiters [Figure 6], whereas no follicular neoplasm showed intraepithelial hemosiderin (*P*<0.0003).

## DISCUSSION

Once a diagnosis of papillary thyroid carcinoma can be excluded on an FNA, the distinction between a benign colloid nodule and a follicular neoplasm remains a difficult problem. Published criteria have emphasized what amounts to be a single criteria - the interdependent features of the size of the follicles, the amount of colloid, and the cellularity. Additional criteria have been suggested in other publications, but quantification of the relative value of these criteria is lacking in the literature. Criteria that appear useful for excluding a follicular neoplasm include variation from follicular group to follicular group, in cytologic features.[6,12]

Another reported criterion is the presence of hyperplastic papillary fragments that lack the morphologic features of papillary thyroid carcinoma.[12,13] Benign colloid nodules occasionally have papillary fragments with fibrovascular cores (best appreciated in cell block sections),[12,13] but such papillary fragments are lacking in follicular neoplasms. Dense sclerosis also seems to favor a benign goiter.[6,12]

One more criterion that has not received attention and appears independent of the previous criteria is the presence of a flattened cell shape in any microfollicles.[12,14] In our experience, any microfollicles encountered in a benign colloid nodule are more commonly lined with a flattened, attenuated epithelium compared to a cuboidal or even slightly columnar epithelium in the microfollicles of follicular neoplasms.[12]

In this study, we quantified whether cystic change, defined as the presence of macrophages, and hemosiderin, either in macrophages or in the follicular cells, distinguish non-Hürthle cell follicular neoplasms from benign colloid nodules.[6,15] An extensive search revealed that these easily quantifiable features have not been validated. Also, they have not been given recent attention for distinguishing benign colloid nodules from follicular neoplasms. Indeed, at a recent national meeting, audience responses to our question about the significance of hemosiderin in thyroid FNAs were mixed.

Our results illustrate that if PTC and Hürthle cell neoplasms are excluded, the presence of hemosiderin laden macrophages strongly supports the diagnosis of benign colloid nodule (*P*<0.0001). Importantly, hemosiderin was not observed in macrophages in any of the 17 follicular carcinomas in this study. Hemosiderin within follicular epithelial cells was a less common finding in FNAs of benign colloid nodules than hemosiderin in macrophages, but it appears particularly useful because it was not found in any of 64 follicular neoplasms.

The frequent hemorrhage and cyst formation in benign colloid nodules likely reflects a fundamental difference in the organization of the vasculature of benign colloid nodules, when compared to follicular neoplasms. A testable hypothesis is that benign colloid nodules have increased venous pressure, as compared to normal thyroid or follicular neoplasms. Increased venous pressure would explain the gross appearance of a bulging, edematous benign colloid nodule, and it explains the microscopic presence of characteristic ‘watery’ colloid. It can also explain the presence of siderophages through the same mechanism as in venous stasis ulcers of the skin or hemosiderin-laden macrophages in bronchial secretions in patients with congestive heart failure. Increased venous pressure could reflect a compression of the veins by the benign colloid nodule itself. As a result of the venous compression, a positive feedback loop would be established, consisting of increased edema and swelling, which in turn leads to further compression and increased venous pressure on the venous system draining the nodule. Follicular neoplasms seem to be able to grow in such a manner that the venous drainage keeps pace with cell proliferation. Noninvasive means of measuring venous pressure (e.g., by Doppler) may be able to test this hypothesis and lead to a useful diagnostic test.

## COMPETING INTEREST STATEMENT BY ALL AUTHORS

No competing interest to declare by any of the authors.

## AUTHORSHIP STATEMENT BY ALL AUTHORS

Each author acknowledges that this final version was read and approved.

According to International Committee of Medical Journal Editors (ICMJE http://www.icmje.org) “author” is generally considered to be someone who has made substantive intellectual contributions to a published study.

Authorship credit should be based on 1) substantial contributions to conception and design, acquisition of data, or analysis and interpretation of data; 2) drafting the article or revising it critically for important intellectual content; and 3) final approval of the version to be published. Authors should meet conditions 1, 2, and 3. Other contributors, who do not meet these criteria for authorship, are listed in an acknowledgments section.

All authors of this article declare that we qualify for authorship as defined by ICMJE http://www.icmje.org/#author.

Each author has participated sufficiently in the work and take public responsibility for appropriate portions of the content of this article.

## ETHICS STATEMENT BY ALL AUTHORS

This study was conducted with approval from Institutional Review Board (IRB) (or its equivalent) of all the institutions associated with this study. Authors take responsibility to maintain relevant documentation in this respect.
